# Developing implementation strategies for the framework and strategy for disability and rehabilitation in South Africa: An approach using the Expert Recommendations for Implementing Change framework

**DOI:** 10.4102/ajod.v15i0.1804

**Published:** 2026-03-10

**Authors:** Naeema A.R. Reis, Sonti Pilusa, Natalie Benjamin-Damons, Juliana Kagura

**Affiliations:** 1 Department of Physiotherapy, School of Therapeutic Sciences, Faculty of Health Sciences, University of the Witwatersrand, Johannesburg, South Africa; 2 School of Public Health, Faculty of Health Sciences, University of the Witwatersrand, Johannesburg, South Africa

**Keywords:** disability policy, rehabilitation services, implementation strategies, framework evaluation, stakeholder engagement

## Abstract

**Background:**

The expiry of South Africa’s Framework and Strategy for Disability and Rehabilitation (FSDR) created an opportunity to critically assess its effectiveness, strengths and limitations to inform the development of a new disability and rehabilitation policy.

**Objectives:**

As the primary gap in the FSDR related to weak implementation rather than policy intent, this study aimed to develop evidence-informed implementation strategies. Findings from a document review, semi-structured interviews and focus group discussions were triangulated and guided by the Expert Recommendations for Implementing Change (ERIC) framework.

**Method:**

A descriptive qualitative design was used. Four focus group discussions were conducted with stakeholders involved in FSDR implementation to reach consensus on practical strategies to strengthen implementation. Data were analysed using combined inductive and deductive thematic approaches, and identified strategies were mapped to the ERIC framework.

**Results:**

Seven themes were identified as key determinants of implementation success or failure: limited awareness and training; resource constraints; poor interdepartmental collaboration; weak governance and policy buy-in; inadequate monitoring and evaluation systems; the need for context-specific approaches; and ongoing professional development. Stakeholders actively contributed to refining strategies within each thematic area.

**Conclusion:**

The findings highlight critical implementation gaps in training, governance, resources and collaboration. Addressing these through targeted, contextually appropriate strategies can strengthen future disability and rehabilitation policy implementation and improve service delivery in South Africa.

**Contribution:**

This study offers a roadmap for improving disability and rehabilitation policy implementation and can inform both future policy planning and clinical service delivery.

## Introduction

Globally, an estimated 15% of the population experiences some form of disability, with South Africa reporting a prevalence of approximately 7.5% (Ding et al. 2022; Lee et al. 2020). Despite progressive policies such as the framework and strategy for disability and rehabilitation (FSDR) aligning with the United Nations Convention on the Rights of Persons with Disabilities (UNCRPD), challenges in effective implementation remain persistent (Hussein El Kout et al. 2022). These barriers, including insufficient resources, weak intersectoral collaboration, and inadequate monitoring mechanisms, significantly hinder the delivery of rehabilitation services (Maart, Mji & Morris 2024).

The FSDR was introduced as a strategic response to promote equitable and efficient disability and rehabilitation services (Maart et al. 2024). However, its implementation has been inconsistent, often impeded by systemic, structural, and operational challenges (Charumbira et al. 2024). These include a lack of awareness on policy implementation among stakeholders, fragmented governance, and resource limitations, all of which exacerbate disparities in access to rehabilitation services (Mudzi 2024). Understanding and addressing these barriers is critical to optimising rehabilitation outcomes and aligning South Africa’s healthcare system with global standards (Blose, Cobbing & Chetty 2021).

The aim of this study was to develop actionable implementation strategies for the FSDR, leveraging the Expert Recommendations for Implementing Change (ERIC) framework (McHugh et al. 2022). By triangulating findings from document reviews, semi-structured interviews, and focus group discussions, this study aims to provide a comprehensive evaluation of the FSDR’s implementation outcomes. The findings may contribute to strengthening disability and rehabilitation policy implementation in South Africa by offering targeted, evidence-informed strategies that enhance the practical uptake and operationalisation of policy within the health system.

This article presents a broad approach to evaluate implementation determinants, propose targeted strategies, and identify key facilitators and barriers. The insights derived from this study aim to inform the development of context-specific, stakeholder-driven solutions, bridging gaps in policy execution and advancing the realisation of the rights and well-being of persons with disabilities.

## Research methods and design

This study focused on the development of implementation strategies to guide future public health policies related to disability and rehabilitation in South Africa. Data on barriers used to map implementation strategies were drawn from an earlier phase of a larger study. This included a document review of FSDR implementation reports (Hussein El Kout et al. 2025a), 15 semi-structured interviews, and six initial focus group discussions from a linked study conducted by the same first author (Hussein El Kout et al. 2025c).

Ethical clearance was obtained from the University of the Witwatersrand Human Research Ethics Committee (Medical). In this study, proposed implementation strategies were presented to stakeholders. For this article, the primary empirical data consisted of four additional focus group discussions, which were conducted to review and refine the proposed implementation strategies until stakeholder consensus was reached. The remaining data: document review findings, interviews, and earlier focus group discussions were drawn from a previous phase of the larger study and were used only to inform and contextualise the strategy development. The focus groups were conducted between August 2024 and November 2024. Stakeholders’ recommendations were used to finalise the strategies. This process adhered to the Partnership, Engagement, and Collaboration (PEC) approach described by Huang et al. (2018), emphasising the critical role of stakeholders’ involvement in implementation science (Hussein El Kout, Pilusa & Benjamin-Damons 2023).

The ERIC framework was used for concept mapping during the design of the implementation strategies. A total of four focus group discussions were conducted to ensure representation across South Africa’s multilevel governance structure. One focus group discussion (FGD) was held at the national level (*n* = 9), one at the provincial level (*n* = 10), one at the district level (*n* = 11), and one with organisations of persons with disabilities (OPDs) (*n* = 8), resulting in 38 participants overall. Groups of 8–15 participants engaged in discussions lasting one to two hours, conducted either in person or online, depending on availability. These groups included rehabilitation programme managers, policy advisers, implementers, health service managers, senior and junior rehabilitation professionals, and OPD representatives. Conducting FGDs across these levels allowed the study to capture variation in implementation experiences, resource contexts, and governance perspectives. The FGDs were conducted until data saturation was reached, where no new themes emerged. In this study, participants were recruited using purposive and snowball sampling, in which initially purposively sampled participants referred other participants to participate in the study. Purposively sampled participants were stakeholders identified as implementers in the FSDR process who had been involved in the implementation of the FSDR at any point between 2015 and 2022 and providing rehabilitation care at any level. Participants included rehabilitation professionals, academics, and representatives from OPDs. A discussion schedule was used to guide each session, supporting interactive dialogue and helping to minimise researcher bias through structured yet flexible engagement. The insights generated during these discussions were triangulated with findings from the document review and earlier phases of the study, enhancing the trustworthiness of the results by allowing different data sources to provide complementary and convergent perspectives. The focus groups were instrumental in reviewing and refining the proposed implementation strategies, ensuring that they reflected stakeholders’ experiences and contextual realities. The final strategies therefore represent a collaboratively developed, evidence-informed, and context-specific approach to strengthening the implementation of disability and rehabilitation policies.

### Data analysis

Maxqda 2020.20 software (VERBI – Software. Consult. Sozialforschung. GmbH, Berlin, Berlin, Germany) was used to analyse the FGD transcripts via inductive and deductive thematic analysis, the document review and interview and focus group discussion transcripts. The software provided a platform for the first author to organise and safely store the codes, themes and subthemes. The ERIC framework was used to map implementation strategies according to determinants identified. Triangulation of data sources ensured comprehensive insights into all aspects of FSDR implementation.

### Ethical considerations

Ethical clearance to conduct this study was obtained from the Human and Research Ethics Committee (Medical) of the University of the Witwatersrand (No. M220364).

## Results

### Participants’ demographics

A total of four FGDs were conducted, bringing together 38 participants across three levels of governance (national, provincial, and district) and OPDs. Stakeholders included rehabilitation programme managers, policy implementers, health service managers, and representatives from OPDs (see Table 1).

**TABLE 1:** Demographic characteristics of focus group participants.

Characteristic	FGD 1 – National Level (*n* = 9)	FGD 2 – Provincial level (*n* = 10)	FGD 3 – District level (*n* = 11)	FGD 4 – OPDs (*n* = 8)	Total (*n* = 38)
**Gender**					
Female	6	7	8	6	27
Male	3	3	3	2	11
Age range (years)	35–60	30–55	28–58	25–50	25–60
Professional role	Policy advisers, programme managers from NDoH	Provincial rehabilitation coordinators, senior therapists (occupational therapists, physiotherapists, speech therapists and audiologists)	District rehabilitation managers, senior and junior occupational therapists, physiotherapists, podiatrists, audiologists	OPD representatives, professional bodies for physiotherapy, occupational therapy and speech therapy and audiology	Multisectoral professionals
Years of experience in rehabilitation	10–25	8–20	5–18	5–15	5–25
Sector representation	National Department of Health	Provincial health departments	District health services	OPDs and professional bodies for rehabilitation	Public and non-governmental sectors

NDoH, National Department of Health; FGD, focus group discussion; OPD, organisations of persons with disabilities.

The FGDs were designed to reflect South Africa’s multilevel governance structure, enabling the study to capture variation in implementation experiences and resource contexts.

### Implementation strategies

**FIGURE 1:**
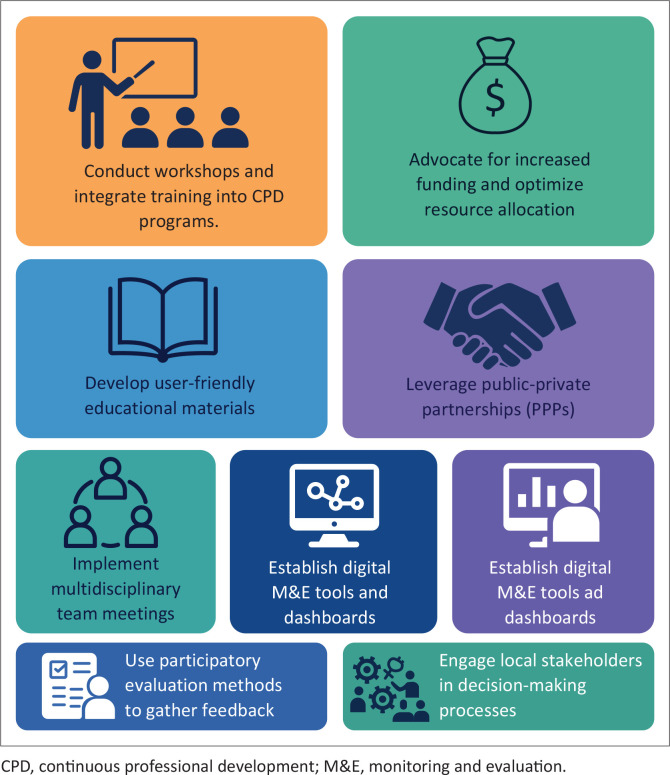
An overview of the implementation strategies.

The key themes, findings, and proposed strategies for improving the implementation of FSDR in South Africa are outlined in Table 2. These strategies aim to address the identified barriers from Hussein El Kout et al. (2025b) and optimise the policy’s effectiveness across various domains. An overview of implementation strategies agreed upon is shown in Figure 1.

**TABLE 2:** An overview of determinants and implementation strategies.

Theme and determinant	Findings	Proposed strategies
Lack of awareness and training Who is not aware and needs training?	Insufficient dissemination and inadequate training on FSDR, leading to poor implementation outcomes	Conduct workshops on policy implementation and integrate this training into CPD programmes Develop accessible content on policy implementation which could be distributed across all levels of care through infographics and digital media
Resource limitations	Staffing shortages, lack of assistive devices, and poor resource allocation hinder service delivery	Advocate for increased funding for rehabilitation workforce and equipment for service provision Leverage public-private partnerships (PPPs)
Need for interdepartmental collaboration Is this at technical level only or at policy level as well?	Siloed operations among health disciplines negatively impact holistic care delivery through the use of the ICF	Implement multidisciplinary team meetings with agendas that demonstrate how holistic care will be achieved and joint case management. Develop digital platforms for shared care planning
Governance and policy buy-in	A lack of leadership support and stakeholder engagement weakens policy implementation	Engage leadership in co-designing policy Provide leadership training on disability-inclusive governance
Monitoring and evaluation What changes do you expect to find?	Absence of thorough M&E systems to track policy implementation and outcomes	Establish digital M&E tools and dashboards Use participatory evaluation approaches with end-users
Context-specific strategies	Diverse needs across districts require localised and adaptive approaches	Develop adaptive strategies tailored to district-specific needs Engage local stakeholders in co-design processes
Professional development	Continuous professional development is essential for sustaining policy implementation	Incorporate policy training into CPD programmes Provide incentives for participation in CPD activities

CPD, continuous professional development; FSDR, framework and strategy for disability and rehabilitation; ICF, international classification of function; M&E, monitoring and evaluation.

The lack of awareness and training on policy implementation among rehabilitation clinicians and other stakeholders, such as OPDs, was one of the key challenges identified in implementing factors for effective implementation of the FSDR. Limited knowledge of the policy’s objectives and guidelines among FSDR policy implementers, as expressed by the participants in the study by Hussein El Kout et al. (2025a), resulted in confusion and inconsistencies in the implementation. The absence of structured training programmes on policy implementation processes further aggravated these issues, leaving staff ill-prepared to integrate the policy into their daily practices. To overcome this, workshops and training programmes focusing on the FSDR’s core components and practical application are recommended. Furthermore, user-friendly educational materials, such as infographics and simplified guides, can ensure accessibility of the policy to staff at all levels. In South African context rehabilitation and disability care are provided through national, provincial, and local systems, as well as private and non-governmental organisations. Staff include professionals such as doctors and rehabilitation therapists, supported by rehabilitation assistants and community health workers.

Another key determinant was equipment and capacity limitations, with stakeholders reporting too few rehabilitation professionals, limited access to assistive devices, and logistical challenges such as a shortage of storage facilities for equipment. Even when these facilities were available, inefficiencies in the distribution and use of resources continued to affect service delivery. Addressing such issues requires not only increased government funding for staffing, training, and equipment but also better coordination, monitoring, and accountability to ensure that resources are used effectively. Working with community organisations and private partners can also help to improve efficiency and reach.

The lack of interdepartmental teamwork further hinders an all-inclusive care delivery. It was reported by clinicians that working in isolation within their respective disciplines, fragmented service delivery and negatively impacted patient care. The proposal was to foster collaboration, multidisciplinary team meetings and joint case management sessions. Further improvements in communications and coordination can be experienced if digital platforms are utilised, enabling the use of shared patient data and collaborative treatment planning. Training programmes emphasising the importance of teamwork and shared care and rehabilitation goal setting can further reinforce interdepartmental collaboration.

A lack of governance and commitment from senior leadership is a major barrier to effective implementation. Stakeholders found that limited involvement and support from management created a gap between policy goals and day-to-day operations, which in turn demotivated frontline staff. Commitment could be enhanced by engaging leadership in co-designing workshops, where they actively contribute to policy adaptation and rollout. Aligning goals and addressing challenges promptly will be achieved if there are leadership training programmes and clear communication channels between managers and staff.

Deficiencies in regular monitoring and evaluation (M&E) mechanisms to track implementation progress and outcomes were highlighted as a significant issue. It is difficult for stakeholders to measure success or to identify bottlenecks without clear performance metrics or feedback loops. Digital tools such as dashboards or mobile apps could provide real-time tracking of policy implementation to address this gap. Regular audits and reporting can ensure accountability, while participatory evaluation processes involving end-users and frontline staff could offer valuable insights from the ground level.

The diversity in resources, infrastructure, and population needs across districts highlighted the need for context-specific strategies. A one-size-fits-all approach was deemed ineffective because of region-specific barriers such as transportation challenges and cultural differences. This may be overcome by developing localised strategies through district-specific consultative workshops and tailor-made solutions to meet unique needs. Sharing successful models and best practices across districts can enable adaptation and duplication in similar situations.

Professional development was emphasised by the stakeholders as the cornerstone for sustaining policy implementation. However, a lack of integration of the FSDR into existing continuous professional development (CPD) activities and limited motivation among staff to engage in such programmes were noted because of the lack of incentives associated with policy implementation. Including the FSDR training in compulsory CPD activities can guarantee regular exposure to the policy. Motivation and incentives, such as certification, promotions, and recognition, can boost participation. Utilising e-learning platforms and virtual workshops for professional development programmes would enhance accessibility and make participation more convenient.

## Discussion

The findings of this study indicated numerous determinants of FSDR policy implementation, including resource constraints, limited governance, and individualised operations as discussed by Hussein El Kout et al. (2025b). By utilising the ERIC framework, this study suggests various options to resolve these challenges. The emphasis on the need for implementation strategies that are stakeholder-driven, context-specific solutions highlights the need for specific application efforts for greater impact. This study reflects critical themes that highlight all the challenges in implementing the FSDR in South Africa. These themes align with current literature on implementation science, rehabilitation policy, and health systems improvements (Bustos, Sridhar & Drahota 2021; Shogren et al. 2020).

Noteworthy determinants of the FSDR implementation included insufficient dissemination of the FSDR policy document and inadequate training on its implementation (Hussein El Kout et al. 2025a). Many clinicians could not effectively adopt the policy because of a lack of understanding thereof. Awareness and competency-based training are critical to successful policy implementation (Morris et al. 2021). Integrating disability-inclusive modules into medical and allied health curricula could strengthen foundational knowledge. Including ongoing professional development specific to the FSDR within health professional training programmes would promote consistent engagement and application of the policy. Workshops, interactive learning modules, and simplified educational materials, such as infographics, could help with understanding the content and would be beneficial for all staff (Anaby et al. 2021).

The stakeholders highlighted resource challenges, including staffing shortages, a lack of assistive devices, and poor resource allocation (Hussein El Kout et al. 2025b). Service delivery is further limited because of logistical issues such as inadequate storage and equipment maintenance, despite available budget allocation, which is consistent with findings by Rauch, Negrini and Cieza (2019). In low- and middle-income countries, such challenges are common, with rehabilitation services often competing with other healthcare priorities. The solution to these constraints requires support for increased funding, efficient resource distribution, and innovative solutions such as public–private partnerships and community-based rehabilitation initiatives (Shogren et al. 2020). Other strategies, such as delegating responsibilities to less specialised staff, can also optimise existing human resources and alleviate staffing shortages.

Siloed operations among health disciplines significantly hindered the holistic delivery of rehabilitation services (Hussein El Kout et al. 2025). Communication and coordination across departments are vital to prevent fragmented care, which negatively impacts patient outcomes (Bustos et al. 2021). It is essential for departments to work together to ensure comprehensive rehabilitation. Multidisciplinary team meetings and joint case management sessions could encourage teamwork and improve care coordination. Digital platforms for shared patient records and collaborative treatment planning could eliminate miscommunication. Structured protocols, shared goals, and accountability mechanisms would further reinforce collaboration and support for integrated care across departments (Shogren et al. 2020).

The lack of leadership support and stakeholder engagement were identified as critical barriers to the successful implementation of the FSDR (Hussein El Kout et al. 2025). This divide between strategic policy objectives and operational execution demotivated the staff and resulted in limited policy adoption (Morris et al. 2021). Governance and leadership are vital for driving the adoption of policies. Involving senior management in the design and implementation of policies would improve their commitment. Leadership training programmes focusing on disability-inclusive governance and establishing clear communication channels between management and staff would further enhance buy-in and accountability, promoting a supportive environment for successful policy execution (Shogren et al. 2020).

A strong monitoring and evaluation (M&E) system was considered a critical requirement according to stakeholders to ensure tracking the progress and impact of the FSDR. Without performance metrics and/or feedback loops, stakeholders are unable to measure success or identify implementation challenges (Rauch et al. 2019). Effective M&E systems are essential for accountability and continuous improvement. Digital tools such as real-time dashboards can assist with data collection and reporting, enabling timely alerts where necessary. Participatory evaluation approaches involving end-users and frontline staff can improve the use of and dependability on the M&E frameworks, providing actionable insights to address bottlenecks and inform decision-making (Shogren et al. 2020).

The diversity in resources, infrastructure, and population needs across districts necessitated specific strategies to identify areas. A one-size-fits-all approach was deemed ineffective in addressing unique regional barriers, such as transportation challenges and cultural differences (Anaby et al. 2021). Specific implementation, adapted to the identified socioeconomic and cultural factors, is more likely to succeed. Engaging local stakeholders in co-designing interventions ensures that solutions are relevant and effective for their specific environments. Sharing successful models and best practices across districts can assist with the adaptation of effective solutions to similar situations (Morris et al. 2021).

The findings of this study highlight the importance of a systems-thinking approach to policy implementation. The identified challenges can be resolved with a multipronged strategy that integrates training, resource optimisation, governance, and stakeholder involvement. Leveraging global frameworks such as the ERIC and Proctor models provides a structured pathway for refining and operationalising implementation strategies (Shogren et al. 2020).

## Conclusion

Disability and rehabilitation-related policy implementation in South Africa faces multifactorial barriers. Focusing on targeted interventions to address the identified barriers of policy implementation may improve the delivery of rehabilitation services in Gauteng and in South Africa, advancing the rights and well-being of persons with disabilities.
